# Synergistic Effects of Chinese Herbal Medicine: A Comprehensive Review of Methodology and Current Research

**DOI:** 10.3389/fphar.2016.00201

**Published:** 2016-07-12

**Authors:** Xian Zhou, Sai Wang Seto, Dennis Chang, Hosen Kiat, Valentina Razmovski-Naumovski, Kelvin Chan, Alan Bensoussan

**Affiliations:** ^1^School of Science and Health, National Institute of Complementary Medicine, Western Sydney UniversityPenrith, NSW, Australia; ^2^Faculty of Medicine, University of New South WalesSydney, NSW, Australia; ^3^School of Medicine, Western Sydney UniversityCampbelltown, NSW, Australia; ^4^Faculty of Medicine and Health Sciences, Macquarie UniversitySydney, NSW, Australia; ^5^School of Pharmacy and Biomolecular Sciences, Liverpool John Moores UniversityLiverpoor, UK; ^6^Faculty of Science, TCM Division, University of TechnologySydney, NSW, Australia

**Keywords:** synergy, Chinese herbal medicine, interaction, isobologram, combination index, system biology

## Abstract

Traditional Chinese medicine (TCM) is an important part of primary health care in Asian countries that has utilized complex herbal formulations (consisting 2 or more medicinal herbs) for treating diseases over thousands of years. There seems to be a general assumption that the synergistic therapeutic effects of Chinese herbal medicine (CHM) derive from the complex interactions between the multiple bioactive components within the herbs and/or herbal formulations. However, evidence to support these synergistic effects remains weak and controversial due to several reasons, including the very complex nature of CHM, misconceptions about synergy and methodological challenges to study design. In this review, we clarify the definition of synergy, identify common errors in synergy research and describe current methodological approaches to test for synergistic interaction. We discuss the strengths and weaknesses of these models in the context of CHM and summarize the current status of synergy research in CHM. Despite the availability of some scientific data to support the synergistic effects of multi-herbal and/or herb-drug combinations, the level of evidence remains low, and the clinical relevancy of most of these findings is undetermined. There remain significant challenges in the development of suitable methods for synergistic studies of complex herbal combinations.

## Introduction

The “one drug, one target, one disease” approach has for some time remained the conventional pharmaceutical approach to the development of medicines and treatment strategies. However, over the last decade, this mono-substance therapy model has gradually shifted toward the adoption of combination therapies, in which multiple active components are employed. This paradigm shift has been partly driven by its limited effectiveness in chronic diseases, treatment resistance, and side effects of synthetic mono-drugs. Recent evidence has demonstrated that combination therapy could provide greater therapeutic benefits to diseases such as AIDS, cancer, atherosclerosis and diabetes, all of which possess complex etiology and pathophysiology and therefore are difficult to treat using single drug target approach (Devita et al., 1975; Chesney et al., 2000; Jukema and van der Hoorn, 2004; Weber and Noels, 2011). Moreover, advances in analytical chemistry and molecular biology methods have broadened our understanding of therapeutic targets of diseases and potential multi-target treatment approaches. Significant progress has been made in the study of synergistic effects of drugs. For instance, a recent study has demonstrated the synergistic effect of the combination of metformin and aspirin in treating pancreatic cancer (Yue et al., 2014). In parallel, research into synergistic interactions of multi-component herbal preparations and their interactions with pharmaceutical drugs has also attracted great interest in recent years.

Traditional Chinese medicine (TCM) has been used over thousands of years for the management of disease, maintenance of health, and prolongation of life expectancy in China and other Asian countries such as Japan and Korea. Chinese herbal medicine (CHM) is a key modality of TCM, in which up to 20 herbs are used in combination in a complex herbal formulation. Substantial progress has been made over the last decades in the study of efficacy and mode of actions of some commonly used herbs and formulations (Lam et al., 2006; Kong et al., 2009). However, evidence to support synergistic effects of multiple herbs and their active components remains controversial. For example, some studies have suggested that meaningful synergistic and therapeutic effects of herbal formulations were unlikely due to the low/extremely low levels of active components existed in the herbs (Williamson, 2001; Danz et al., 2002). This has led to skepticism that herbal therapies are merely placebo effects (Tausk, 1998). In contrast, numerous studies have demonstrated that herbal extracts as a whole and/or multiple herbs in complex formulations offer better efficacies than equivalent doses of individual active ingredients and/or herbs when used alone, highlighting the significance of synergistic action in herbal therapies (Leonard et al., 2002; Scholey and Kennedy, 2002; Zhang et al., 2014). In this article, we aim to present a comprehensive review of the current status of research on the synergistic effects of CHM and how they are effectively measured.

## Definition of synergy

Generally, synergy is defined as the interaction of two or more agents to produce a combined effect greater than the sum of their individual effects (van Vuuren and Viljoen, 2011). In medicinal research field, however, the understanding of synergy is complicated. Spinella (2002) has classified the concept of synergy broadly into two main categories based on the mode of actions—pharmacodynamic and pharmacokinetic synergy (Spinella, 2002). The first type of synergy describes two or more agents that work on the same receptors or biological targets that result in enhanced therapeutic outcomes through their positive interactions. The second type of synergy results from interactions between two or more agents during their pharmacokinetic processes (absorption, distribution, metabolism and elimination) leading to changes of the agents quantitatively in the body and hence their therapeutic effects (Spinella, 2002).

The concept of synergy is an intrinsic part of TCM philosophy and is often described in a more holistic way. The complex synergistic interactions among the herbs in complex CHM formulations (Fufang) are believed to be able to enhance the bioavailability of active components, promote therapeutic effects, and/or reduce toxicity (Jia et al., 2004). The design of herbal formulations follows the principle of compatibility, called “Peiwu,” which requires the considerations of different interrelationships of herbal ingredients including synergism (Xiang Xu), assisting (Xiang Shi), detoxication (Xiang Sha and Xiang Wei), antagonism (Xiang Wu), and rejection (Xiang Fan) (Jia et al., 2004). Based on this principle, different herbs are combined following “Jun-Chen-Zuo-Shi” (also known as “Emperor–Minister-Assistant-Courier”) rule to achieve desirable effects and/or to minimize side-effects. “Jun” is the main herb in a herbal formula with a relatively higher ratio directly targeting the disease; “Chen” is an adjuvant herb to promote therapeutic effect of the key herb or to target the accompanying symptoms; “Zuo” is usually used for reducing the side-effects of the herbal formula; “Shi” is the herb that guides the active ingredients to reach the target organs or to harmonize their actions.

It is important not to confuse synergistic effect with additive effect. Synergy occurs when two or more drugs/compounds are combined to produce a total effect that is greater than the sum of the individual agents (Chou, 2010; Breitinger, 2012), while an additive effect is an add up of individual effects where each individual agent is not affecting the other (no interactions). The concept that the additive effect of two drugs is their “arithmetic sum” is a misconception. The additive effect is not simply the sum of the effects of A plus B. For instance, if agents A and B exert an inhibitory effect as 60 and 70% at certain dosage level, respectively, it is incorrect to state their additive effect is 130% at this dosage. The additive effect needs to be determined using a more complex mathematical algorithm equation.

Comparing combination activity to individual activity at the same dosage or effect level is a common error in synergy studies. A large amount of papers were found to report synergistic effect when the ED_50_ value of a combination was significantly lower than that of each individual component with a *P* < 0.05. This approach seems logical, but it actually cannot distinguish between an additive effect and a synergistic effect as it is still the comparison between combination and individual effects. Similar errors are very common in animal and clinical trials where it is difficult to apply rigorous synergy determining methods due to practical reasons (e.g., costs, curation of the study, ethical issues, etc). In these studies, researchers estimated synergism by simply comparing the effect of the combination with that of individual components, which unfortunately would lead to a wrong conclusion. Thus, more efficient and practical models need to be developed to determine synergistic effects of multi-component preparations in animal and human studies.

## Methods to quantify synergy

Currently, no unified methodology is available for synergy research to facilitate the different understandings of the mode of action of synergy; this has led to the question that whether the drug interactions observed are genuine synergistic effects (Chou, 2010). However, several methodological approaches have been developed and are used for combinational drug therapies. Loewe additivity and Bliss independent are the two oldest and major reference classes for synergy studies in the last few decades (Tang et al., 2015). Loewe additivity is usually applied for the case where the drugs have similar modes of action on the same target or pathway (pharmacokinetic) (Tang et al., 2015). In contrast, the Bliss independence model is expected to hold truth for non-interacting drugs that elicit their responses independently (pharmacodynamic) (Tang et al., 2015). In recent years, many other methods based on these two models have been developed and adapted for the study of synergistic effects of multi-component preparations. Some of the most popular models including the combination index and isobole method (both derived from Loewe additivity), systems biology analysis (used for multi-target synergy), and several specific assays for synergistic studies of antimicrobial agents are discussed here. The strengths and limitations of these methods are summarized in Table 1.

**Table 1:** **Comparison of current models of synergy methods**.

**General methods**	**Brief description**	**Strengths**	**Limitations**
Combination index	A scientific term to quantitatively depict synergism (CI < 1), additive effect (CI = 1), and antagonism (CI > 1).	1) One of the most practical methods experimentally. 2) The most demonstrative method for the proof of synergy effects. 3) No limitation for the number of ingredients in the tested combination.	1) Must be able to determine dose-response of individual constituents and combination.
Isobole method	A graphical procedure that can either represent additive, synergistic, or antagonistic interactions, depending on the position of thc dose of combination to the “iso-effect” linear line.	1) The oldest and well-established method. 2) One of the most practical experimentally.	1) Must be able to determine dose-response of individual constituents and combination. 2) Generally, only applicable for two drug combination.
Systems biology	A computational and mathematical modeling for predicting and understanding the network of components and protein/gene targtes binding biological system.	1) Suitable for study of synergy of multi-components, prodrugs, and novel targets. 2) Being able to investigate the mechanisms of action of a combination, and identify the key active components.	1) Large data sets including chemical, chemogenomics, pharmacological data and the compounds' potential targets information are required.
**Methods specific in microbiology**	**Brief description**	**Strengths**	**Limitations**
Diffusion assays	Positive/negative interactions in the mixture are observed via comparing the bacteria growing inhibition zone diffused in the agar with that of individual agent.	1) Impact on microorganism can be investigated *in vitro*. 2) Simply, visual, qualitative representation of synergistic (or antagonistic) effect of individual components used together.	1) These assays are subject to many variables which may influence the results and should at the most be used as a qualitative guide only. 2) Cannot differentiate synergism from additive effect.
Checkerboard array	The combination of two agents is contructed on a in two dimension array, and the positive/negative interactions are determined by comparing the combinational and individual inhibitory activity which can be quantified by fractional inhibitory concentration (FIC).	1) Clear visualization on a single plate of contribution of the individual components. 2) Can test multiple concentrations simultaneously. 3) Easy to carry out and interpret	1) Assessment of viability is not always accurate when replying on turbidometric readings. 2) Laborious for combination of three, not feasible for combinations of four or more. 3) Rely on a linear dose-response curve for all components. 4) All plants in the combination tested required to be at equal ratios.
Time-kill assay	Positive/negative interactions among multi-components in the mixture are determined via comparing individual and combinational bacteri cidal activity over a series of time intervals.	1) One of the best methods to study synergy of antimicrobial agents. 2) The bacterial cidal effect is monitored over time which is not possible with the frequently used MIC assays.	1) The method is labor intensive and requires a number of steps where variables may be introduced. 2) Difficult in interpretation of results because relatively few antibiotic concentrations are examined. 3) Rely on the reading at one time point (usually 24 h) as the sole determinant of the interaction.

### Combination index

Recently it has been demonstrated that synergy follows physicochemical mass-action law, which briefly states that the ratio between the concentration of reactants and products is constant for a chemical reaction mixture that is in equilibrium. From mass-action law, a simplified mathematic equation for the combination index (CI) was further developed for the quantitative determination of synergy in multiple agents acting on the same target/receptor (Chou, 2010). Synergism occurs when the CI value is < 1 (the more the CI value approaches 0, the stronger the synergistic effect); additive effect occurs when the CI value is equals to 1, and antagonism happens when the CI value is >1.

The CI is a practical model used for the analysis of synergy of a multi-component combination in a fixed ratio. To calculate the CI, the dose-response curves (inhibitory effect, stimulatory effect, etc.) of individual components A and B and their combination in a fixed ratio are determined using the same assay. The doses of each individual component that achieves a specified effect (e.g. 50%, ED_50_) are determined. Further, the doses of A and B required in the combination to produce the same level of effect can be calculated. Then, based on the doses, the synergism/addition/antagonism is calculated according to Equation (1) below for two agents and Equation (2) for multiple agents.

**Figure 1:**
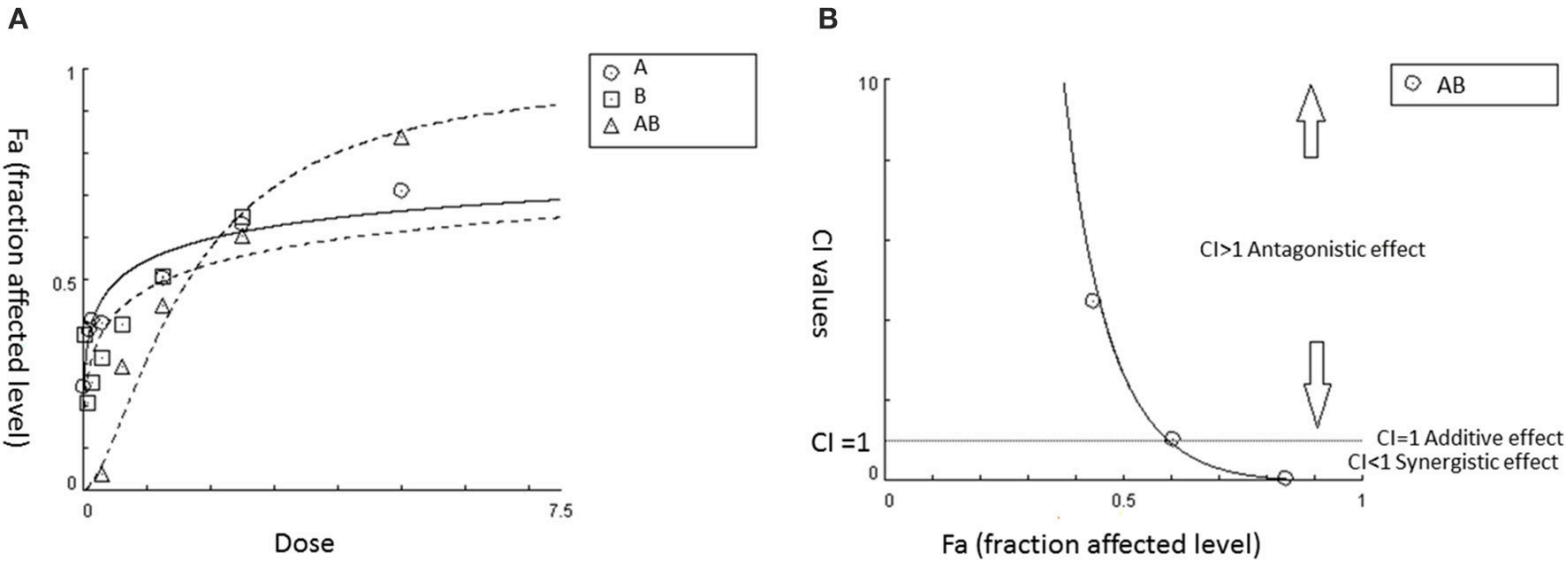
**An example of utilizing CI model to determine synergy for the combination of agent AB in certain fixed ratio. (A)** Dose-effect curves for A, B, and AB, respectively. **(B)** CI value-Fa (Fa: fraction affected level) curve for AB generated from CalcuSyn based on the dose-response curves shown in **(A)**. It demonstrated that synergistic effect is starting from 60% effective level (Fa = 0.6) and this synergistic effect continues to increase (CI < 1) at higher effect levels in AB.

In addition, computer software including “CalcuSyn” and its third-generation “CompuSyn” have been developed, which greatly facilitates the CI analysis (Patrick Reynolds and Maurer, 2005; Chou, 2006). By inputting the dose-effect data of agents 1 and 2 and their combination, the software can generate a Fa (effect level)–CI curve, which demonstrates a complete dataset of CI values (represent antagonistic/additive/synergistic interactions) at all tested doses. Also, a CI-Fa (effect levels) can be generated (an example is shown in Figure 1). This model can also be applied for the determination of interactions among multiple agents in a mixture. The calculation is based on Equation (2), which demonstrates n-drug combination at x% inhibition. Examples using CI model for the determination of synergistic interaction in CHM are discussed in Section Synergistic Interactions within Single Herb Analyzed by CI or Isobologram Method.

(1) $$\begin{array}{cc} \text{CI }=\frac{D1}{d1}+\frac{D2}{d2} & \;(1) \end{array}$$

(2) $$\begin{array}{cc} \left(\text{CI}\right)n\text{x}=\sum_{j\;=\;1}^{n}\frac{\left(D\right)j}{\left(d\right)j} & \;(2) \end{array}$$

^n^(*CI*)x = combination index for n drugs at x% inhibition

*D*1 and *D*2 = doses of individual constituent alone, required to produce a chosen effect level (usually ED_50_)

*d*1 to *d*2 = the doses of individual constituent in the combination required to produce the same effect, respectively.

It is worth mentioning that many studies also reported a synergistic effect when certain pharmacological effects can be observed with a drug combination, but not with one or more of its individual components. This effect is classified as potentiation or augmentation rather than synergism based on Chou's theory (Chou, 2010). If an individual component does not generate an effect when used alone, the D1 value is equals to 0, thus the synergy cannot be determined in the CI equations. This potentiation or augmentation effect can be considered as a form of complementary interactions.

### Isobole method

**Figure 2:**
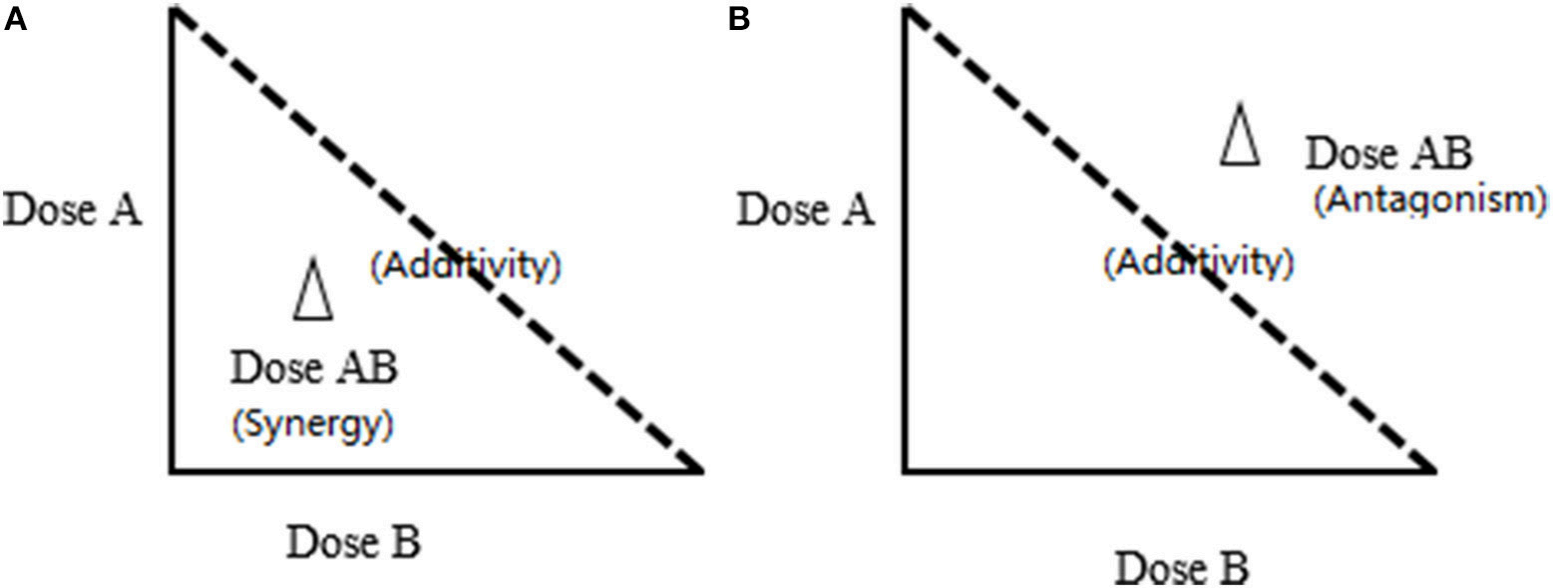
**The lsobole method for synergy study**. Dose A and Dose B are the individual concentrations of Components A and B; Dose AB are the concentrations of A and B in the combination. The dashed line shows zero interaction between A and B, which represents a simple additive effect. The effect of the combination equals the sum of the effects from individual components. **(A)** Effect of synergy: the dot is underneath the dashed line. **(B)** Effect of antagonism: The dot is above the dashed line; Addition: The dot is on the dashed line.

Isobologram, was first introduced by Fraser in 1870 based on Loewe additivity (Fraser, 1872). This method has been widely accepted as one of the most practical models in terms of experimental design and effectiveness to illustrate the synergistic/additive/antagonistic interactions. Similar to the CI model, the isobole method requires the determination of dose-response relationship of the combination and its individual components independently to assess if synergism exists. This is expressed as a dose response curve on an isobole graph as shown in Figure 2. The isobole is an “iso-effect” curve, in which a combination of components (A or B) at different dose levels is represented on the graph, the axes of which are the dose-axes of the individual constituent (A and B). The dashed line joining the points (i.e. ED_50_ of A and B) which represent the same dosage required from the combination as the sum of the individual component to reach the same effect. If the dots of the combination falls on this dashed line, it represents an additive effect, i.e., no interactions between components A and B. If synergy occurs the curve becomes “concave.” The opposite applies for antagonism representing by a “convex” isobole (Figure 2). It is also possible to have synergy at one dose combination and antagonism at another, with the same substances and this would give a complicated isobole with a wave-like or even elliptical appearance.

In theory, isobologram and Fa-CI plot are both based on the same CI equation and therefore should yield an identical conclusion. However, isobologram explains synergy from a dose-oriented perspective (reaching the same therapeutic activity but with a lower dose level required), while Fa-CI plot from an effect-oriented perspective (resulting in a higher therapeutic activity at the same dosage level). Additionally, Fa-CI plot can be used to determine multi-component interactions, whereas isobologram is only feasible for a two drug combination as it is not possible to construct multidimensional isobologram curves.

Both CI and isobologram models are designed for evaluating the synergistic/antagonistic interaction between two or more single-entity agents acting on the same target/receptor. Even though these models have been recently used for analyzing possible synergistic interactions in simple herbal formula (herb-pair), they are by and large less adequate for evaluation of the complex interactions among multiple bioactive components of CHM formulations (Gu and Chen, 2014) (for more detailed discussions see Section Current Status of Synergy Research in Chinese Herbal Medicine).

### Systematic analysis/system to system (S2S)

The CI and isobologram methods both require combination-by-combination evaluations of individual components in a mixture specifically acting on the same target/receptor. However, for a complex combinational therapy especially TCM multi-herb formulations, which often contain hundreds of potentially bioactive components that may act on a network targets, it is technically impossible to use these two models to evaluate all combinations of bioactive components on each of the target one by one.

A systematic analysis (or S2S) approach integrating literature, experimental data, and computational sciences has been recently developed to address the multi-target synergistic actions. To conduct this analysis, the three-dimensional structures of individual compounds in the combination are obtained from relevant database, handbooks or literature, and the known crystal structures of all the key genes/proteins associated with targeted disease are retrieved from relevant data banks or databases. The network constructions between agents and targeted protein/genes are then constructed *via* a molecular docking approach. Molecular docking is usually applied for computer-assisted drug design, which predicts the predominant binding mode(s) of a ligand with a protein of known structure. Here, it is used to analyse binding modes between the potential active compounds and their corresponding target proteins based on their structure binding modes, in order to generate a drug-target network. The predicted interaction between bioactive and targeted receptors from the generated network can either be confirmed by literature or validated through experimental studies (Wang X. et al., 2012; Leung et al., 2013). This model investigates the multi-target mechanisms of action of multi-constituents mixtures and identifies the key active components, which can bind to most of the corresponding targets. In addition, this method can be used in drug development through selecting and combining the most active components that act on the maximum amount of the protein or gene targets. This minimal effective composition with definite constituents and controllable quality can optimize the composition of the mixture while maintaining their curative effects (Li et al., 2012; Liang et al., 2012).

The S2S approach has gained popularity as a promising and valuable tool to evaluate synergy of complex herbal formulations (Lee, 2015). However, due to the poor understanding of the chemical and pharmacological properties of bioactive constituents of many TCM herbs, the application of this method in studying synergistic effects of TCM herbs to gain insight of the holistic approach of CHM remains a challenge (Wang X. et al., 2012).

### Models used in microbiology studies

In microbiology, several bioassays and mathematics models have been developed and applied to the investigations of the synergistic anti-bacterial effects of individual bioactive components of Chinese herbs and antibiotic agents including diffusion assay, checkerboard array, and time-kill assay.

#### Diffusion assay

This is a simple, visual and qualitative microbiology assay that can be used to investigate the combinational effect of individual components used together. Inhibition/kill studies in agar or broth, microscopy and molecular analysis are used to investigate the mode of action of antimicrobial agents. In this method, each individual test samples (A or B) and their combination (A+B) are placed in separate wells or disc on agar containing pathogenic organisms, and their inhibition zones are compared. If the inhibition zone of the combination is larger than that from A or B, positive interactions are noted. Should the inhibition zone of the combination be smaller than A or B independently, then negative interactions are noted. Since it only compares the inhibition zone of combination with individual component, it cannot differentiate synergism from additive effect. Moreover, this method is subject to many variables which may influence the results and is used as a qualitative guide only.

#### Checkerboard array

This method is used for evaluating synergistic interactions of antimicrobial agents against bacteria or fungi. In this method, the effects of interactions are assessed through the serial dilution of two agents A and B [two-fold dilution to four times of estimated minimum inhibitory concentration (MIC); Hsieh et al., 1993). A checkerboard is constructed in a two dimension array with the same amount of series dilutions of agent A and agent B in x and y-axis. Thus, each tube/well in the middle of checkerboard contains a unique combination of agent A and B being tested. With the addition of cell viability dyes such as resazurin or MTT, the inhibitory activity of individual, and the combination of components can be visually assessed and quantified by fractional inhibitory concentration (FIC). In addition, the turbidity of the wells prior to and after incubation with the microbe can be assessed for their difference, which can then also be used as an indicator of MIC. This method is easy to carry out and interpret the interactions between two agents, and multi-concentrations can be tested simultaneously. However, factors such as compound/herb solubility in the wells, biofilm formation, and interactions between the compounds/herbs with the indicator dyes, all of which can be problematic for the turbidometric and/or colorimetric readings, can result in the inaccurate assessment of viability of the test microbe.

#### Time-kill assay

This method provides descriptive information on the relationship between bactericidal activity and the concentration of a test substance. The principle involves exposing the inhibitor to a selected pathogen and, at selected time intervals, aliquots are sampled and serially diluted. The dilutions are plated out, incubated at optimum conditions for the test organism, and the colony for forming units (CFU) are counted and plotted logarithmically against time. Depending on the curve of the dose response, either an additive, synergistic, or antagonistic effects is determined. Since this method can monitor the bactericidal effect over different time-points, it is quite labor intensive and requires a number of steps where variables may be introduced. It is difficult to interpret results because relatively few antibiotic concentrations are examined.

## Current status of synergy research in chinese herbal medicine

For the last decades, it is well-accepted that combined drug therapies may provide better clinical outcomes in the treatment of some conditions such as hypertension, cancer, depression and HIV infection. Synthetic pharmaceutical drugs are usually single chemical entities acting on a single biological target. Combined drug therapies are formulated in a fashion to augment the total effects in treating the target condition, reduce the side effect via dose-sparing of the active components, or address different metabolic interdependence, mediators or risk factors of diseases through a variation of independent biomolecular targets. However, due to the limitation of the methodology currently available, it is still challenging to identify the best possible combinations from the large amount of the existing drugs, evaluate their molecular interactions, and establish their efficacy and safety (Orloff, 2005).

**Figure 3:**
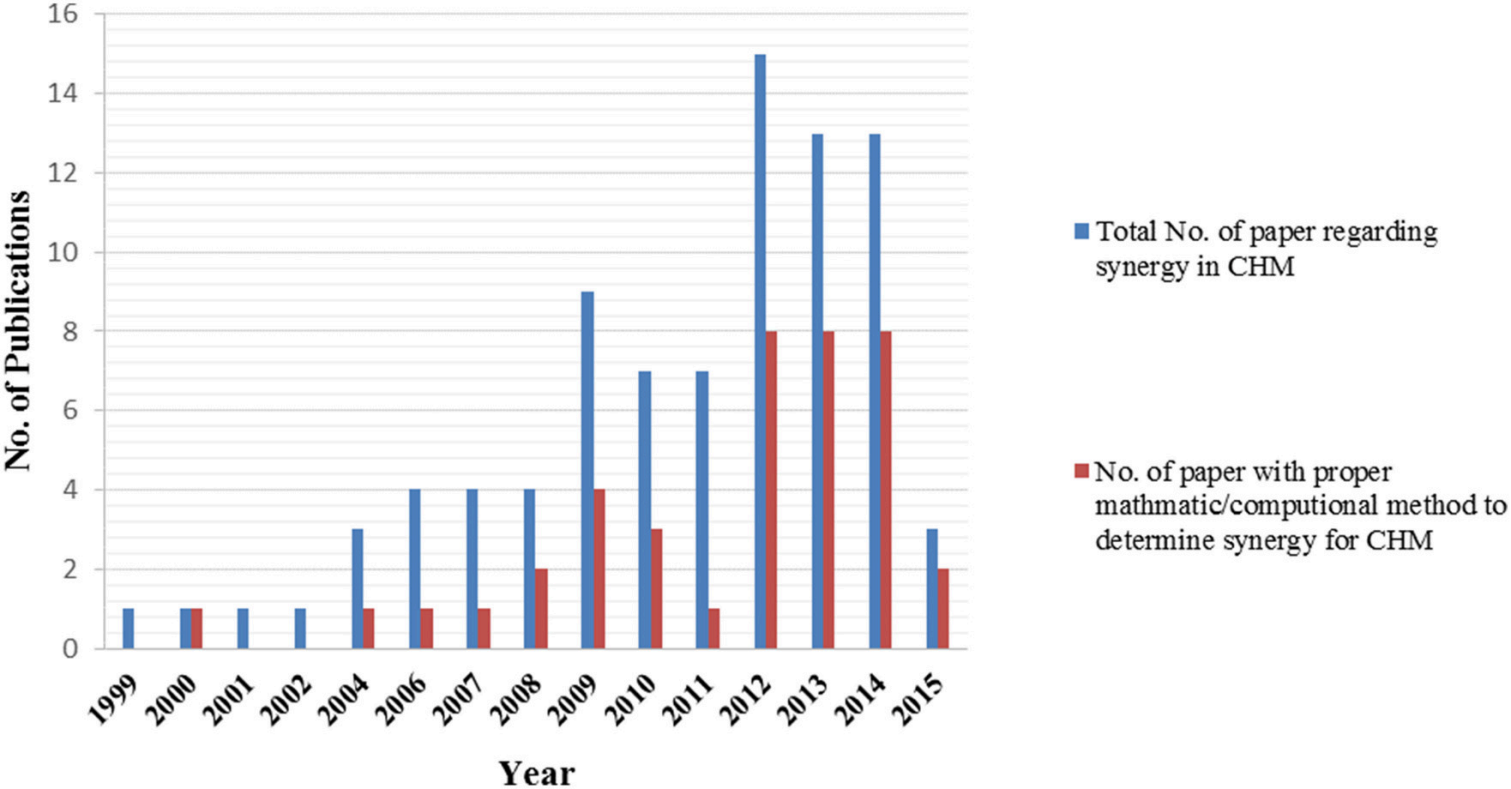
**Histogram showing the general increase in synergy of Chinese herbal medicine related publications in the years 1999–2015 (up to June only for 2015) from a bibliographic search in PubMed and Google Scholar database carried out in June 2015**.

In contrast to western style combined drug therapies where chemical properties and pharmacological effects of individual compounds are well-defined, Chinese herbal formulations are constructed according to TCM theories (“Peiwu” and “Jun-Chen-Zuo-Shi”). Despite the long history of clinical use and a solid theoretical basis, the multi-component and multi-target nature of CHM poses a huge challenge to the study of the mechanisms of action, including synergistic effects underpinning the complex herbal formulations (Wang et al., 2009). With the advance in mathematic modeling (e.g., CI, isobologram) and computer technology (e.g., systems biology analysis), there has been a growing number of systemic and mechanistic studies over the past two decades aiming to provide better scientific evidence and understanding of synergistic effects of Chinese herbal formulation. In this study, we conducted a thorough literature search (keywords used: “Synergism” or “Synergy” or “Synergistic” and “Chinese herbal medicine” or “Chinese medicinal herbs”) in PubMed and Google scholar from the period of 1990 to 2015. The numbers of studies using proper synergy models are shown in Figure 3. Key studies identified are summarized in Table 2.

**Table 2:** **A summary of ***in vivo*** and ***in vitro*** studies on Chinese herbal extracts or active ingredients that have been reported to produce synergistic effects on various biology activities**.

**Method of synergy determination**	**Study type**	**Interacting components**	**Therapeutic activity**	**Evidence of synergy**	**References**
Combination index	*In vitro*	Escin and cisplatin	Anti-cancer	Escin (10 and 15μM) combined with cisplatin (3μM) resulted in a significant synergistic (CI = 0.256 and 0.186) cytotoxic effect in Panc-1 cells.	Rimmon et al., 2013
	*In vitro*	*Astragalus membranaceus* and *Paeonia lactiflora*	Anti-cancer	Seven substances were identified from active fraction combination which represents the synergistic effect for two herb combination on anti-oxidant activity.	Xu et al., 2014
	*In vitro*	*Camptotheca acuminate* and its component camptothecin plus cisplatin	Anti-cancer	Both *Camptotheca acuminate* extract (0.01 to 5 mg/mL) and its isolated compound camptothecin (0.05 to 1 μM) demonstrated a synergistic effect (CI < 1) in the HEC-1A and HEC-1B cells when combined with cisplatin (1–50 μM).	Lin et al., 2014
	*in vivo*	Polyphyllin I (a steroidal saponin extracted from Rhizoma of *Paris polyphyllin*) and evodiamine (a kind of alkaloid from *Evodia rutaecarpa*)	Anti-cancer	Combination of Polyphyllin I (200μg/mL) and platinum (20μg/mL), evodiamine (200μg/mL) and platinum (20μg/mL), evodiamine (20μg/mL), and 5-FU (300μg/ml) had higher inhibition rates than any single drug of them (CI<1).	Yue et al., 2013
	*In vitro*	*Astragalus membranaceus* and* Cimicifuga foetida*	Anti-cancer	The combination of isoferulic acid and calycosin (isolated from each herb) at a dose ratio of 1:1 resulted in significant synergy (CI_50_ = 0.77) in scavenging DPPH radicals and ferric reducing antioxidant power (FRAP) assay. This combination also exhibited synergistic effect at a dose of 1:1 (CI= 0.442) and 2:1 (CI= 0.636) in HepG2 cell-based assay.	Wang et al., 2014
	*In vitro*	Four anti-proliferative phytocompounds in *Wedelia chinensis*	Anti-cancer	Four bioactive compounds were identified in *W. chinensis* to inhibit androgen receptor activity in prostate cancer cells and the four active compounds acting together exerted strong synergism (CI= 0.39–0.78).	Lin et al., 2007
	*In vitro*	*Mentha piperita* L. and *Salvia officinalis* L.	Anti-cancer	At 31.25, 62.5, and 125μg/mL dosage levels, cancer cells treated with *Mentha piperita* L. plus *Salvia officinalis* L. combinations (1:1) showed significantly lower viability than calculated values based on individual extracts [CI= 0.67± 0.09 (<1)].	Yi and Wetzstein, 2011
	*In vitro*	Xanthorrhizol (isolated from *curcuma xanthorrhizza*) and curcumin (not stated for the source)	Anti-cancer	Synergistic activity (CI< 1) was commenced from the combination xanthorrhizol-curcumin 3:7 to 1:9 to induce MDA-MB-231 cells death.	Cheah et al., 2009
	*In vitro*	*Scutellaria baicalensis*, *Rabdosia rubescens*, *Panax-pseudo ginseng*, *Dendranthema morifolium*, and *Glycyrrhiza uralensis*, and Serenoarepens	Anti-cancer	*S. baicalensis* and *D. morifolium* when combined (equipotent quantities and at 1/2 and 1/4 fractions of their IC_50_ value) were additive with a trend toward synergy, whereas *D. morifolium* and *R. rubescens* together were additive. The remaining two-extract combinations showed antagonism. The four extracts together were significantly more effective than the two-by-two combinations and the individual extracts alone.	Adams et al., 2006
	*in vivo*/*in vitro*	Realgar-Indigo naturalis formula (RIF): tetraarsenic tetrasulfide (from realgar), indirubin (from *Indigo naturalis)*, and tanshinone IIA (from *Salvia miltiorrhiza)*	Anti-cancer	Tetraarsenic tetrasulfide, indirubin and tanshinone IIA at concentrations ranging from 0.25 to 1μM had CI values<1, indicating synergic effects on human APL cell differentiation.	Wang L. et al., 2008
	*In vitro*	“Chong Lou Fu Fang” (CLFF)–Rhizoma Paridis, Fructus Forsythiae, and Radix Codonopsis plus 5-fluorouracil (5-FU) (chemotherapeutic agents)	Anti-cancer	The synergistic analysis indicated that CLFF (0.05–0.35 mg/mL) with 5-FU (0.75–75.25μM) had a synergistic cytotoxicity effect in a relative broad dose inhibition range (20–95% fraction affected in SGC-7901 cell lines and 5–65% fraction affected in BGC-823 cell lines), while the synergistic interaction between CLFF and oxaliplatin or docetaxel only existed in a low dose inhibition range (≤50% fraction affected in both cell lines).	Liu et al., 2011
Interaction index	*In vitro*	ASHMI formula and its components: aqueous extracts of Lingzhi (*Ganoderma lucidum*), Kushen (*Sophora flavescens*), and Gancao (*Glycyrrhiza uralensis*)	Anti-asthma	By comparing the interaction index values, constituents in ASHMI (individual extracts in the percentages of 35, 45, and 20 of Lingzhi, Kushen and Gancao) synergistically inhibited eotaxin-1 production as well as Th2 cytokine production.	Jayaprakasam et al., 2013
Median –effect analysis/Combination index	*In vitro*	Three main phthalides from *Angelica sinensis*: n-butylidenephthalide (BLP), senkyunolide A (SKA) and z-ligustilide (LGT)	Anti-cancer	Three main *Angelica sinensis* phthalides (the composition ratios identical to that in the extracts of *Angelica sinensis* and Ligusticum chuanxiong, respectively) in *Angelica sinensis* extract yielded a synergistic effect (CI< 1) whereas Ligusticum chuanxiong extract exerted an antagonistic effect (CI> 1) on the inhibition of cell proliferation in colon cancer cells at the tested doses.	Kan et al., 2008
Combination index and isobologram	*In vitro*	*Strobilanthes crispus* (SCS) and tamoxifen	Anti-cancer	The combined SCS (8.5μg/ml or 10.0μg/ml) and tamoxifen (2.5 to 15μM) treatment displayed strong synergistic inhibition in MCF-7 (CI= 0.32–0.40) and MDA-MB-231 (CI= 0.29–0.52) cell growth at low doses of the antiestrogen.	Yaacob et al., 2014
	*In vitro*	Rhizoma Corydalis and Rhizoma Curcumae	Anti-cancer	A combination of two herbal extracts exhibits the strongest anticancer cell proliferation effect at the ratio of 3:2 (ezhu to yanhusuo)	Gao et al., 2009
Isobologram	*In vitro*	Curcumin (extracted from the rhizomes of Curcuma species) and NVP-BEZ235	Anti-cancer	Combined treatment of NVP-BEZ235 (0.5–4 mM) and curcumin (30μM) demonstrated synergistic effects on apoptosis in human renal carcinoma Caki cells.	Seo et al., 2014
	*In vitro*	Berberine (from *Coptis chinensis* Franch.) and evodiamine (from *Evodiae Fructus*)	Anti-cancer	Berberine (0–0.1μM) and evodiamine (0–0.18μM) mixture showed the highest inhibition effect (50.00%) as compared with berberine and evodiamine used individually (20.24 and 16.33%, respectively) to induce apoptosis on human hepatocellular carcinoma SMMC-7721 cells over 48 h.	Wang X. N. et al., 2008
“System to system” (S2S) mode/System biology	*In vitro*/*In vivo*	*Panax ginseng* and* Salvia miltiorrhiza*	Myocardial ischemia	An optimized minimal phytochemical composition (new formula NSLF6) were achieved from Pananx ginseng-*Salvia miltiorrhiza* combination which has activity for treatment MI through synergistic therapeutic efficacies between total ginsenosides and total salvianolic acids via promoting cardiac cell regeneration and myocardial angiogenesis, antagonistic myocardial cell oxidative damage.	Liang et al., 2012
	*In vivo*/*In vitro*	Compound *Danshen* formula (*Salvia Miltiorrhiza*, P*anax Notoginseng* and Borneol*)*	Cardiovascular diseases	Radix Salviae Miltiorrhizae (*Danshen*) tackled 39 out of total 41 validated CVD targets (except eNOS and ACE2) which acted as the emperor (key herb) in this formula, whereas Panax Notoginseng interacted with 36 potential targets in which 34 overlapped with *Danshen*'s targets and serve as minister and courier drugs.	Li et al., 2012
	*In vivo*/*In vitro*	Radix Astragali Mongolici, Radix Puerariae Lobatae, Radix Ophiopogonis Japonici, and Radix Salviae Miltiorrhiza	Cardiovascular diseases	The structural properties of molecules in four herbs have substantial differences, and each herb can interact with significant target proteins related to CVD. Moreover, the bioactive ingredients from different herbs potentially act on the same molecular target (multiple-drug-one-target) and/or the functionally diverse targets but with potentially clinically relevant associations This study has demonstrated that multiple-drug-multiple-target-one-disease therapeutic pattern of TCM formula.	Wang X. et al., 2012
	*In vivo*	*Panax ginseng* (PG) and *Salvia miltiorrhiza* (SM)	Myocardial ischemia	SLF (SM: PG= 3:7) exerted synergistic therapeutic efficacies to exhibit better effect on MI compared to PG or SM.	Liang et al., 2011
	*In vivo*/*In vitro*	Radix curcumae formula: Radix Curcumae, Fructus Gardeniae, Moschus, and Borneolum	Cardiovascular diseases	The pharmacological system generated 58 bioactive ingredients from the Chinese herbal Radix Curcumae formula, and predicted 32 potential targets related to the CCVD. The results indicates that Radix Curcumae share the most common targets with Fructus Gardeniae (15), while less common targets with Moschus and Borneolum (8 and 1, respectively). Further integrated network shows that Radix Curcumae represents the principal component for the prevention of CCVD, and other three medicines serve as adjuvant ones to assist the effects of the principal component, which together probably display synergistic actions.	Tao et al., 2013
	*In vivo*	Yinchenhao decoction: *Artemisia annua* L. *Gardenia jasminoids* Ellis, and *Rheum Palmatum* L.	Hepatic injury syndrome (HI)	Three major active compounds combination DGR [6,7-dimethylesculetin (D), geniposide (G), and rhein (R)] from Yinchenhao decoction, exerts a more robust therapeutic effect than any one or two of the three individual compounds by hitting multiple targets in a rat model of hepatic injury.	Wang et al., 2013
	*In vivo*	Yin-Chen-Hao-Tang (YCHT): *Artemisia annua* L. (the monarch herb), Gardenia jasminoides Ellis (the minister herb), and *Rheum Palmatum* L. (the assistant and servant herb): 6,7-Dimethylesculetin (D), geniposide (G) and rhein (R) were extracted from those herb ingredients, respectively	Hepatic injury syndrome (HI)	DGR combination could increase the plasma level, slow elimination rate, exert a more robust therapeutic effect than any one or two of the three individual compounds by hitting multiple targets in a rat model of HI.	Zhang et al., 2011
Checkerboard dilution method	*In vitro*	Pseudolaric acid B (Herbal source was not stated in the paper) and fluconazole	Anti-fungal	FICI showed a synergism of pseudolaric acid B and fluconazole (0128 to 0.125μg/mL) against azole-resistant clinical isolates of *C. albicans*, whereas there was no such reaction with other Candida species.	Yan et al., 2012
	*In vitro*	Glabridin (from the root of *Glycyrrhiza glabra*) and 17β-E2	Estrogenic effect	When glabridin was treated together with 17β-E2 (1:1), synergistic estrogenic effect was observed with a slight decrease in cell proliferation as compared to treatment by 17β-E2 alone.	Su Wei Poh et al., 2015

### Synergy reflected in chemical fingerprint

Chemical fingerprint studies utilizing high-performance liquid chromatography chromatography, gas chromatography, liquid chromatography–mass spectrometry and chemometric resolution methods have been conducted to detect changes in the amount of pharmacologically active compounds in individual herbs when given in combination (Han et al., 2007; Wang et al., 2011; Kaliappan et al., 2014). During the decocting preparation, the chemical composition is often altered due to solvent's polarity, heating effects or changed pH environment (Li, 2014). These changes in chemical composition could account for the increased therapeutic effects and/or reduced side effects of the individual herbs. Li et al. (2006) demonstrated that the amount of main volatile chemical components present in Rhizoma Ligustici Chuanxiong (*Chuanxiong*) and Radix Paeoniae Rubra (*Chishao*), which are commonly used to promote blood circulation and remove stasis, were markedly altered when given together due to the formation of non-volatile, water soluble salts during the decoction process (Li et al., 2006). In addition, the amount of aconitine, a toxic component of *Aconitum carmichaeli* Debx. (*Fuzi*) is significantly reduced due to its chemical reaction with tannine under high temperature.

Chemical fingerprint studies have also shown that new compounds are formed when herbs are given in combination during the preparation. For instance, Wang Y. L. et al. (2012) identified four new compounds in a Radix Polygalae (*Yuanzhi*) and Acorus Tatarinowii Rhizoma (*Shichangpu*) combination that were not detected in the individual extracts (Wang Y. L. et al., 2012). Although, the biological effects of these new compounds were not evaluated in this study, it provides possible explanations for the changes in pharmacological properties when herbs are used in combination.

### Synergistic interactions within single herb analyzed by CI or isobologram method

Individual herbal extracts consist of a complex mixture of bioactive compounds, concomitant agents and other minor substances among which interactions can occur leading to synergistic effects. For example, *Ginkgo biloba* extract has been shown to possess an anti-platelet aggregation property (Dutta Roy et al., 1999), which could contribute to its therapeutic effects for vascular cognitive impairment through enhancing cerebral blood flow (Zhang and Xue, 2012). Synergistic interaction between ginkgolides A and B, two bioactive components of *Ginkgo biloba*, has been demonstrated in a platelet aggregation test using the isobole method (Williamson, 2001).

In a study conducted by Lin et al. (2007), the anti-cancer property of four H_2_O sub-fraction phytocompounds (indole-3-caroxyaldehyde, wedelolactone, luteolin, and apigenin) from *Wedlia chinensis* were evaluated using three different androgen receptor (AR)-dependent cancer cell lines (human PCa22 Rv1, 103E, and LNCaP cells) (Lin et al., 2007). Interestingly, as revealed by the CI analysis using CalcuSyn software, a significantly stronger anti-cancer effect was observed when the four compounds were combined at the same ratio as in the original herb extract when compared with that of each individual compound highlighting the synergistic property of these compounds present in *Wedlia chinensis*. It is worth mentioning that the anti-cancer activity was significantly reduced when any one of these bioactive compounds was removed from the combination, indicating the significance of the four bioactive compounds acting as a whole.

Kan et al. (2008) demonstrated the anti-cancer activities of the three major phthalides mixture [butylidenephthalide (BLP), senkyunolide A (SKA), and z-ligustilide (LGT)] from *Angelica sinensis* with the same ratio as they were in the extract on colon cancer HT-29 cell line. Although three phthalides mixture demonstrated synergistic cytoxic effects, it was shown that the anti-proliferative effects of the whole *A. sinensis* extract was still significantly higher than of the corresponding phthalide mixture. Therefore, it suggested positive interactions of active components and other co-existing components within the herb (Kan et al., 2008).

### Synergistic interactions within multi-herbal combinations

#### Synergistic interactions in herb-pair analyzed by CI or isobologram method

Multiple herbs are commonly used in Chinese herbal formulations, and pose further methodological challenges in synergy research. *Salvia miltiorrhiza* (*Danshen*) and *Pueraria lobate* (*Gegen*) are commonly used in combination for the treatment of coronary heart disease (Sieveking et al., 2005; Koon et al., 2011; Ng et al., 2011). A study conducted by Cheung et al. (2012) demonstrated that the anti-atherogenic effects of Danshen-Gegen combination in a 7:3 (w/w) ratio produced synergistic, additive and antagonistic effects in anti-inflammation, anti-foam cell formation, and anti-vSMC proliferation, respectively. This was the first study demonstrating the feasibility of applying CI analysis in the synergy study of herbal combinations (Wing Shing Cheung et al., 2012).

Several other studies have been conducted to investigate synergistic interactions of two-by-two combinations in complex formulations. For example, synergistic effects of five commonly used medicinal herbs extracts—thyme (*Thymus vulgaris*), rosemary (*Rosmarinus officinalis*), sage (*Salvia officinalis*), spearmint (*Mentha spicata*) and peppermint (*Mentha piperita*) were tested in an *in vitro* study (Yi and Wetzstein, 2011). In this study, individual extracts were paired at 1:1 ratio and their anti-cancer activities were compared in SW-479 colon cancer cell line using CI model by CalcuSyn software. The sage and peppermint combination (1:1) produced the strongest and synergistic inhibitory effects on cancer cell growth at the doses ranging from 31.25 to 125 μg/mL when compared to that of individual single extracts and other combinations (CI value of 0.67 ± 0.09). Similarly, Adams et al. investigated the interactions among five popular herbs (*Scutellaria baicalensis, Rabdosia rubescens, Panax-pseudo ginseng, Dendranthema morifolium, Glycyrrhiza uralensis* and *Serenoarepens*) in 22Rv1 cell line using the isobolographic analysis and revealed that the effects of the four extract combination were significantly greater than that of individual extracts alone and the two-by-two combinations (Adams et al., 2006).

#### Identifying active chemical constituents contributing to synergistic interactions

In recent years, a new methodology that combining CI model and fractionation technique was developed for identifying and isolating synergistic interacted active constituents. For example, in a study by Xu et al. (2014), synergistic anti-oxidant activities of *Astragalus membranaceus* (AME) and *Paeonia Lactiflora* (PL) combination were reported. In this study, AME and PL extracts residues were combined and different fractions were obtained by further sequential extraction with different solvents. Their anti-oxidant effects were then tested using free radical scavenging assay (DPPH), and total phenolic and flavonoid contents assays. The fraction combination which showed synergistic effect by CI model was selected for further fractionation. Eventually, the optimized fraction producing the strongest synergistic effect was subjected to HPLC-MS/MS analyses and seven components were successfully identified which contributed to the anti-oxidant activity of AME-PL combination including oxypaeoniflora, catechin, calycosin-7-O-b-D-glucopyranoside, fomononetin-7-O-b-Dglucopyranoside, 9,10-dimethoxy-pterocarpan-3-O-b-D-glucopyranoside, quercetin, and 29-dihydroxy-39,49-dimethyl-isoflavan-7-O-b-D-glucopyranoside (Xu et al., 2014). A similar study was conducted by Wang et al. (2014) in a *Radix Astragali* and *Cimicifuga foetida* combination (Wang et al., 2014). The most potent fraction combinations were identified by comparing different fractions from each herb and their combinations using DPPH and ferric ion reducing antioxidant power (FRAP) assays. Several bioactive components were identified by HPLC-ESIMS/MS including calycosin, and formonoetin from *Radix Astragali*, and ferulic acid and isoferulic acid from *Cimicifuga foetida* (Wang et al., 2014). These studies have clearly demonstrated a relatively efficient method to isolate and identify active compounds from herbal combination, providing an exemplar for mechanistic study of synergistic effects of CHMs. However, it is worth pointing out that the fractionation method employed in these two studies discarded the “inactive” fractions and therefore, their possible interactions with the active compounds were not tested/considered. Indeed, it has been demonstrated that the antioxidant effects of the active components isolated from *Salvia plebeian* R. Br. were much weaker than that of the crude extracts, indicating that the components “inactive” may play a role in the pharmacological effects of the herbal combinations (Gu and Weng, 2001).

#### Systematically determine synergistic interactions within multi-herb formulations

“System to system (S2S)” or “systematic analysis” methods integrating chemomic and systems biology have recently been employed in the study of synergistic effects of complex herbal formulations. This method analyses the multi-target actions of mixed chemical constituents with a system of targeted protein/receptors [see Section Systematic Analysis/System to System (S2S)], thus it is more suitable for the complex nature of TCM formula. For example, Liu Wei Di Huang Wan is a classic TCM formula used to manage various complex diseases such as hypertension and esophagus carcinoma. It consists of 6 herbal ingredients including Shu Di Huang (Rehmanniae Radix Praeparata), Shan Zhu Yu (Corni Fructus), Mu Dan Pi (Moutan Cortex), Shan Yao (Dioscoreae Rhizoma), Fu Ling (Poria), and Ze Xie (Alismatis Rhizoma), each of them were found to have multiple pharmacological effects. Liang et al. (2014) attempted to evaluate the mechanisms underlying the interactions of hundreds of chemical constituents in the formula on their potential biological targets by utilizing a novel network pharmacology approach. It was found that the key synergistic interactions occur on multiple systems including maintaining homeostasis in endocrine system, immune system and metabolism. However, the significance of this study is limited due to the fact that not all potentially bioactive ingredients from the formula were identified and that their pharmacological properties were thoroughly defined. Nevertheless, the results have demonstrated the holistic mode of action of a herb formula targeting on a network based multi-systems, and highlighted the difficulties associated with synergistic research in CHM (Liang et al., 2014).

Li et al. (2012) attempted to illustrate the synergistic mechanisms underpinning the “Jun-Chen-Zuo-Shi” theory of CHM in a study of *Danshen* formula (CDF) using a systems-pharmacological network model. CDF is commonly used for CVDs consisting of *Danshen* (*Radix Salviae Miltiorrhizae*) as the key (“Jun”) herb, *Sanqi* (*Panax Notoginseng*) as the adjuvant (“Chen”) herb, and Borneol (*Borneolum*) as the courier (“Shi”) herb. In this study major bioactive compounds that have high oral bioavailability from each herb were screened and selected through a robust *in silico* model OBioavail 1.1. Based on the available pharmacological data of these candidate compounds, their potential targets were searched through “PharmMapper Server,” and those targets that were related to the pathological processes of CVD were selected. Thus, the compound-target network was constructed, and the interactions/relationship of the compounds from each herb work on the same or different targets for CVD was investigated. The results demonstrated that candidate compounds from *Danshen* tackled 39 out of total 41 validated CVD targets (except eNOS and ACE2), whereas that from *Sanqi* interacted with 36 potential targets, 34 of which overlapped with *Danshen*'s targets. Furthermore, the network analysis showed that the compounds from *Danshen* targeted the whole CVD systems, whereas that of *Sanqi* placed emphasis on the modulation of vascular smooth muscle cells providing evidence to support the former as the “Jun”/key herb and the latter as the “Chen”/adjuvant herb in the formula (Li et al., 2012). In a similar study, the underlying cardio- and cerebrovascular protective mechanism of a complex formula consisting of *Radix Curcumae* (*Yujin*), *Fructus Gardeniae* (*Zhizi*), *Moschus* (*Shexiang*), and *Borneolum* (*Bingpian*) was assessed by network reconstruction method through matching bioactive components with good oral bioavailability with their potential targets on cardio- and cerebrovascular system. The data showed that 58 bioactive compounds identified from the herbs in the formula were shown to interact with 32 potential targets relating to cardiovascular and cerebrovascular diseases (CCVD). It was suggested that *Radix Curcumae* was the key herb in this formula as it contained the most candidate compounds that possessed high degree distribution on CCVD related targets (8 targets) followed by *Fructus Gardeniae* as the minister herb (5 targets), Moschus (2 targets) as the adjuvant herb and Borneolum (0 targets) as the messenger herb (Tao et al., 2013).

In addition, systems biology can also be used to screen the active components from the complex formula that act on the major therapeutic targets. Shuanglong formula (SLF), consisting of *Panax ginseng* and *S. miltiorrhiza*, is a popular Chinese herbal formulation for the treatment of myocardial infarction (MI) (Liang et al., 2012). Liang et al. (2012) employed a S2S method using cell lines, isolated tissue and animal models to optimize the phytochemical composition of SLF and revealed that the cardiac protective effect of SLF is primarily through synergistic interactions between ginsenosides and salvia acids from *Panax ginseng* and *S. miltiorrhiza*, respectively (Liang et al., 2012). Based on these results a new formulation, NFSL6 consisting total ginsenosides and total salvianolic acids at ratio of 7:3 was successfully developed using pharmacological screening. Similarly, Wang et al. (2013) applied systematic analysis in the study of Yinchenhao decoction (consisting *Artemisia annua* L.*, Gardenia jasminoids Ellis*, and *Rheum Palmatum* L.), a classical formula used for treating hepatic injury syndrome. Three major active components including 6,7-dimethylesculetin (D), geniposide (G) and rhein (R) were found to be the main contributors to the efficacy of the formula and the D-G-R combination synergistically reduced histologic changes and hepatocyte apoptosis, reversed the relevant metabolic biomarkers, and modified 15 target protein expressions in the rat hepatic injury model (Wang et al., 2013).

These studies have demonstrated that the “system to system” or “systems-pharmacological network” models are applicable to the study of synergistic effects in complex herbal formulations. The results appear to support the rationality of “Jun-Chen-Zuo-Shi” theory of complex Chinese herbal formulations and promoted the understanding of the multi-components interactions in these formulations. In addition, by exploring the active fractions/components from a formula, it facilitates the development of new formulations and/or new drug candidates. However, these methods reply heavily on the availability of the chemical and pharmacological data concerning the active components in some well-studied herbs/herbal formulas and therefore are not applicable to many herbs or herbal formulas in which their active components unknown and molecular targets unclear.

### Synergistic interactions between chinese herbal medicine and pharmaceutical drugs

Due to the rapid expansion of the concurrent use of pharmaceutical medicines and Chinse herbal medicine, research into their interactions and the underlying mechanisms is urgently needed. Some of these interactions may be therapeutically beneficial via synergism to enhance therapeutic effects or via antagonism to reduce side effects. Most of the synergistic studies available relate to anti-cancer therapies. For example, anti-cancer properties of Polyphyllin I (PPI–active component from Rhizoma of *Paris polyphyllin*) and Evodiamine (EVO–active component from *Evodia rutaecarpa*) are well-documented (Lee et al., 2005; Adams et al., 2007; Chan et al., 2009; Kong et al., 2010), but their efficacy is generally weaker than that of chemotherapeutic agents such as platinum (Pt), 5-Fluorouracil (5-Fu), and irinotecan (CPT-11). However, when PPI or EVO were combined with Pt or 5-Fu, it produced a significantly stronger inhibition rate than Pt or 5-Fu alone on freshly-removed gastric cancer tissues from patients. The CI values were found to be <1 in all combinations at fraction affected (Fa) = 80%, indicating a synergistic anti-cancer effect (Yue et al., 2013).

Lin et al. (2014) demonstrated that the combination of aqueous extract of the leaves and fruit of *Camptotheca acuminate* (AE-CA) and cisplatin (platinum analogs) exerted synergistic cytotoxicity effects in HEC-1A and HEC-1B human endometrial carcinoma cells. This study also demonstrated that the cytotoxic effect of the mixed AE-CA extracts was significantly stronger than that of camptothecin (CPT), a chemotherapeutic drug isolated from *C. acuminate*. However, the AE-CA extracts produced similar effects on cell-cycle regulation and the accumulation of cyclin-A2 and -B1 as CPT. Therefore, the existence of synergistic effects among the individual components in *C. acuminate* extracts remains unclear (Lin et al., 2014).

Similarly, the synergistic effects of bioactive dichloromethane (DCM) subfraction of *Strobilanthes crispus* leaves (SCS) and tamoxifen (antiestrogen drug) were investigated using estrogen receptor-responsive and non-responsive breast cancer cells (MCF-7 and MDA-MB-231 cell lines). SCS extracts alone induced around 50% cell death after 24 h treatment. When combined with low doses of tamoxifen (which did exert any significant cytotoxic effect on its own), SCS extracts produced 80% cell death after 24 h treatment, which was proved to be a synergistic effect based on the method of CI using Calcusyn software (Yaacob et al., 2014).

A study by Seo et al. (2014) has shown that a mixture of curcumin, a main bioactive component of *Curcuma longa* and NVP-BEZ 235 (a potent dual inhibitor of PI3K and mTOR) can synergistically induce apoptosis in human renal carcinoma caki cells (Seo et al., 2014). The mixture of 2 μM NVP-BEZ235 and 30 μM curcumin significantly provoked cell shrinkage, membrane blebbing, chromatin damage in the nuclei and DNA fragmentation; none of these effects were as significant when curcumin and NVP-BEZ235 were used alone. Isobologram analyses demonstrated that there were synergistic activities between curcumin and NVP-BEZ 235.

### Synergistic interactions between chinese herbal medicine and antibiotics in microbiology studies

Synergistic effects between antibiotics and herbal medicine have also been extensively studied. Agar diffusion assay is often used for screening the anti-bacterial activity of individual agent, and used as qualitative guide for positive/negative interaction guide for agents' mixture. For example, four active constituents (pseudolaric acid B, gentiopicrin, rhein and alion) extracted from CHMs (herbal source was not stated in the paper) were tested on agar diffusion assay, and only pseudolaric acid B showed significant fungicidal activity with growth inhibition zones ranging from 8 to 25 mm against *C. albicans, C. glabrata, C. krusei, C. tropicalis, C. dubliniensis* and *C. parapsilosis*. Furthermore, a positive interaction was observed between pseudolaric acid B and fluconazole in the agar diffusion assay on the same disc, where there was no microcolony growth in the cross-sectional area of pseudolaric acid B and fluconazole observed (Jiang et al., 2010). In addition, checkerboard array and time-kill assay are commonly used methods to quantitively determine the synergism/addition/antagonism interactions. Jang et al. (2014) explored the synergistic antibacterial activities of baicalein, a flavonoid originally isolated from the root of *S. baicalensis* Georgi, with ampicillin and/or gentamicin against a range of oral bacteria strains. Their results showed that both baicalein-ampicillin and baicalein-gentamicin combinations demonstrated synergistic bactericidal effects evidenced by FICI ≤ 0.5 determined by checkerboard array (Jang et al., 2014). In addition, time-kill array results confirmed the positive interaction between baicalen–ampicillin and baicalen–gentamicin, respectively, which showed substantially stronger bactericidal effect in combination compared with individual effect from 0 to 24 h (Jang et al., 2014). Similarly, Hwang et al. (2013) also utilized checker-board array to analyse the synergistic effect between amentoflavone (biflavonoid class of flavonoids, isolated from *Selaginella tamariscina*) and antibiotics such as ampicillin, cefotaxime and chloramphenicol. Their results showed that amentoflavone had a considerable antibacterial effect and exerted synergistic interactions with antibiotics against all bacterial strains tested (FICI ≤ 0.5) except for Streptococcus mutans. This synergistic effect was suggested to be largely mediated by the formation of hydroxyl radical by nicotinamide adenine dinucleotide phosphate (NADH) depletion (Hwang et al., 2013).

## Conclusion

In this review, we have clarified the definition of synergy from two angles pharmacodynamics (enhanced therapeutic actions on the same target) and pharmacokinetics (no direct interaction but with multi-target behavior), as well as elucidated the common misconceptions of synergy. It is critical to distinguish between synergistic effects and simple additive effects of individual herbs or active ingredients in a complex herbal formulation. Several rigorous analytical methods such as isobolographic analysis, combination index (CI), and systems biology have been developed for the quantitative evaluations of synergistic effects. These methods have greatly facilitated the development and standardization of combination therapies (e.g., chemotherapy treatment) in conventional western medicine. However unlike western medicine where the chemical and pharmacological properties of individual drugs are clearly defined, herbal medicine often contains numerous active ingredients, which can all contribute to their synergy effects. This poses a huge challenge in the study of synergistic effects of herbal medicine and a different research approach is needed to accurately evaluate and quantify these effects.

We have also summarized current available methods for the study of synergistic effects of CHM. These methods have been successfully used to determine the nature (i.e., synergistic, additive, or antagonistic) of the interactions within single herbs, multiple herbal formulations or between pharmaceutical drugs and herbal medicine. Each of these methods has strengths and weaknesses and therefore careful consideration should be given when designing a synergistic study of herbal medicine. For example, the CI and isobologram models are relatively fast and simple and are ideal for studying the interactions of a small number of active components or herbal extracts in which their chemical and pharmacological properties are well-defined. However, the CI or isobologram methods are designed for mono-target therapies, and it is of limited use in demonstrating synergistic effects in complex combined therapies especially those in CHM. S2S mode/systems biology methods approach the issues from a systematic perspective, which is consistent with the holistic approach of Chinese medicine. This network system may be more suited for the study of synergistic mechanisms underlying complex herbal formulations. However, these methods rely on the availability of large amount of chemical and pharmacological data, which are absent at the moment for most of the herbs and herbal formulations. More research into the chemical and molecular/pharmacological bases of herbal medicine and their bioactive components is needed before these analyses can be applied more broadly.

In summary, synergy research on CHM is still at its infancy. The currently available methods have various significant limitations on the synergy studies of CHM; more methodological development is urgently needed in the future. Despite synergistic effects demonstrated in numerous pharmacological studies, these findings do not always represent the clinical therapeutic superiority. Therefore, the clinical benefits of multi-component combinations must be subsequently confirmed in rigorous clinical trials.

## Author contributions

AB conceived the study. AB and DC designed the study. XZ and SS searched all the relative papers and drafted this manuscript. DC, HK and AB helped to revise the manuscript. All authors read and approved the final manuscript.

### Conflict of interest statement

The authors declare that the research was conducted in the absence of any commercial or financial relationships that could be construed as a potential conflict of interest.
